# Efficient unfolding pattern recognition in single molecule force spectroscopy data

**DOI:** 10.1186/1748-7188-6-16

**Published:** 2011-06-06

**Authors:** Bill Andreopoulos, Dirk Labudde

**Affiliations:** 1 Department of Bioinformatics, Biotechnological Center, University of Technology Dresden, Dresden, Germany; 2 Department of Bioinformatics and Computer Science, University of Applied Sciences Mittweida, Mittweida, Germany

**Keywords:** protein unfolding, single-molecule force spectroscopy, pattern recognition, Force-Distance curve

## Abstract

**Background:**

Single-molecule force spectroscopy (SMFS) is a technique that measures the force necessary to unfold a protein. SMFS experiments generate Force-Distance (F-D) curves. A statistical analysis of a set of F-D curves reveals different unfolding pathways. Information on protein structure, conformation, functional states, and inter- and intra-molecular interactions can be derived.

**Results:**

In the present work, we propose a pattern recognition algorithm and apply our algorithm to datasets from SMFS experiments on the membrane protein bacterioRhodopsin (bR). We discuss the unfolding pathways found in bR, which are characterised by main peaks and side peaks. A main peak is the result of the pairwise unfolding of the transmembrane helices. In contrast, a side peak is an unfolding event in the alpha-helix or other secondary structural element. The algorithm is capable of detecting side peaks along with main peaks.

Therefore, we can detect the individual unfolding pathway as the sequence of events labeled with their occurrences and co-occurrences special to bR's unfolding pathway. We find that side peaks do not co-occur with one another in curves as frequently as main peaks do, which may imply a synergistic effect occurring between helices. While main peaks co-occur as pairs in at least 50% of curves, the side peaks co-occur with one another in less than 10% of curves. Moreover, the algorithm runtime scales well as the dataset size increases.

**Conclusions:**

Our algorithm satisfies the requirements of an automated methodology that combines high accuracy with efficiency in analyzing SMFS datasets. The algorithm tackles the force spectroscopy analysis bottleneck leading to more consistent and reproducible results.

## 1 Introduction

Mutations cause structural instabilities in a protein leading it to misfold. The misfolded protein conformation may interrupt ion transport and signal transduction. Protein instability and misfolding cause disease states, including cystic fibrosis, Charcot-Marie-Tooth disease, arrhythmias, hearing loss and retinitis pigmentosa [1].

**Figure 1:**
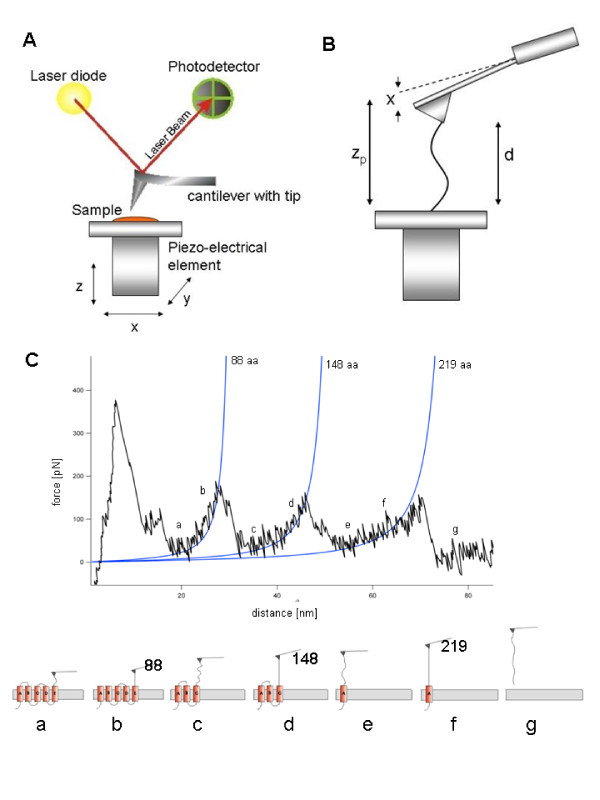
**Unfolding of a membrane protein: a single molecule is attached between the tip of a cantilever and the sample, while a force is applied to unfold and stretch the protein**. The resulting Force-Distance (F-D) curve indicates protein unfolding. The force peaks are fitted by the Worm-Like Chain (WLC) model and are correlated with unfolding of the protein's secondary structure elements (amino acids). The force peaks are related to energy barriers, i.e., energetically favored regions of the protein structure [13].

The number of protein structures deposited each year in the Protein Data Bank (PDB) has quadrupled over the past decade. However, the exact structures of many proteins remain unsolved due to the practical difficulties in the crystallization process for X-ray crystallography or resolving structures with NMR [2]. In the last decade the single-molecule force spectroscopy (SMFS) method was established for experimental investigations on proteins (membrane and globular) and cells [3,4]. During continuous stretching of a protein, the applied forces are measured by the deflection of the cantilever and plotted against extension, yielding a characteristic Force-Distance (F-D) curve, as Figure 1 shows. With the help of automated robots, repeated SMFS experiments can be performed on a protein, resulting in thousands of individual F-D curves. Each F-D curve exhibits a specific pattern, which contains information about unfolding pathways and stable intermediates, and their probabilities of occurrence when unfolding the protein. For membrane proteins the sequence of observed unfolding peaks follows the amino acid sequence of the protein. Fitting a peak in the F-D curve to the Worm-Like Chain (WLC) model or another model (such as, the freely jointed chain (FJC), or the freely rotating chain (FRC)), gives us the number of already unfolded amino acids in the protein (contour length). With the peaks and the known secondary structure, it is possible to associate the unfolding events to the structural domains [5,6].

To distinguish F-D curves showing different protein unfolding pathways, and draw statistical conclusions on the unfolding events' locations (amino acids), their occurrences, and their co-occurrences with other events, one must be able to analyse a large number of F-D curves by objective procedures [7]. The manual analysis is known to be slow and subject to human errors [8]. There is a need for data analysis and pattern recognition algorithms that offer fully automated processing of large SMFS datasets on the basis of objective criteria [9]. The scientific analysis of F-D curves should reveal the molecular interactions and different unfolding pathways. So far, various software packages have been developed to analyze SMFS data [10-12]. In this paper, we propose an algorithm for an automated classification and analysis of F-D curves. We apply and evaluate our method on a dataset of unfolding experiments performed on the bacterioRhodopsin (bR) membrane protein.

## 2 Biological datasets

### 2.1 Structure of the bacterioRhodopsin trimer/lipid complex

**Figure 2:**
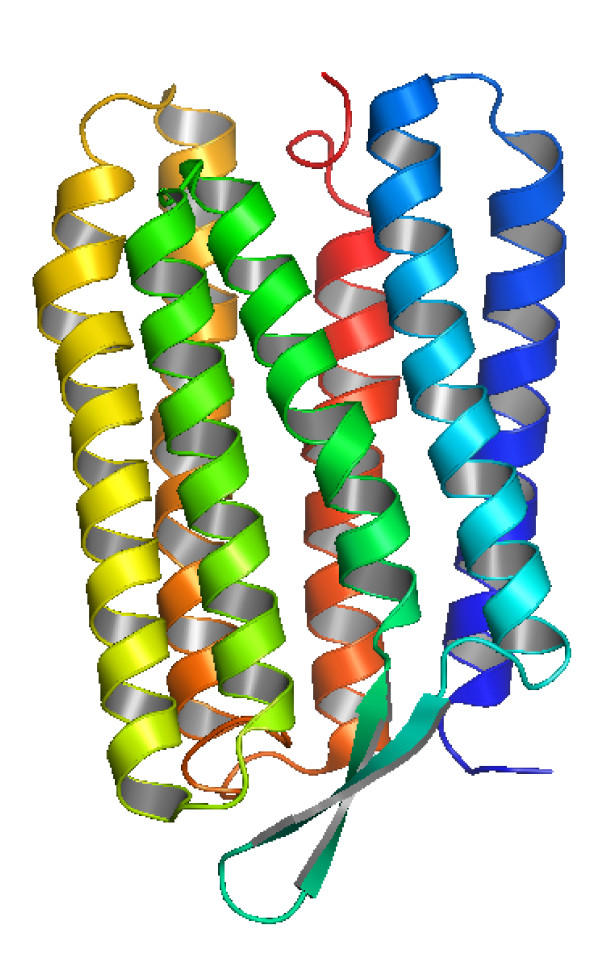
**The 3D structure of bacterioRhodopsin (the structural model PDB:**1BRR**)**. F and G helices are blue; D and E helices are green; B and C helices are yellow; A helix is red. [29]

The light-driven proton pump bacterioRhodopsin (bR) was chosen as a model system for this study because it represents one of the most extensively studied transmembrane proteins. bR converts the energy of light into an electrochemical proton gradient, which in turn is used for Adenosine Triphosphate (ATP) production by the cellular ATP synthase [5]. The part of bR that traverses the membrane usually consists of seven helices. Transmembrane helices are usually about 20 amino acids in length. Figure 2 shows the seven helices in bR in perpendicular views [13]. The helices are connected by loops that are exposed to the aqueous environment on either side of the membrane and that, therefore, consist of residues with polar side chains [14-16]. The bR helices are lettered A, B, C, D, E, F and G, starting from the N-terminus and ending at the C-terminus [17].

Figure 1 shows that the maximum rupture length of the unfolded bR molecule would be 92 aa (~ 29 *nm*) if the tip binds to the CD loop, and 158 aa (~ 50 *nm*) if the tip binds to the AB loop; the last potential barrier would be built by the G-helix. By selecting the F-D curves exhibiting an overall length between 180-220 aa (~ 60 - 70 *nm*) we are sure to analyze only curves from bR molecules that were attached by their C-terminus to the SMFS tip [16,18].

### 2.2 Analysis of bR unfolding pathway

To evaluate the quality and performance of our method, we used a dataset on the bR protein including 26 F-D curves. Our goal is the detection of possible unfolding pathways in bR [19-21]. Figure 1 shows a typical F-D curve. The force (pN) is either output by the AFM or it is computed by multiplying the cantilever deflection (nm) with the spring constant (pN/nm). The distance is the tip-sample separation (nm) between the cantilever tip and the sample surface (the length of the extended protein); this is either output by the AFM or else it is computed by subtracting the deflection from the Z-sensor (nm).

The main unfolding pathway of bR is characterised by the presence of three main peaks, which suggest a pairwise unfolding of the transmembrane helices [22]. On manual analysis of bR unfolding pathways it was found that besides three main peaks that occur in most F-D curves, other peaks referred to as side peaks occur with smaller probabilities indicating that bR can exhibit different unfolding intermediates. The goal of our algorithm is to match the peaks between different curves if they correspond to the same unfolding events; then, unfolding pathways can be distinguished on the basis of unfolding events.

## 3 Methodology for Force-Distance pattern recognition

**Figure 3:**
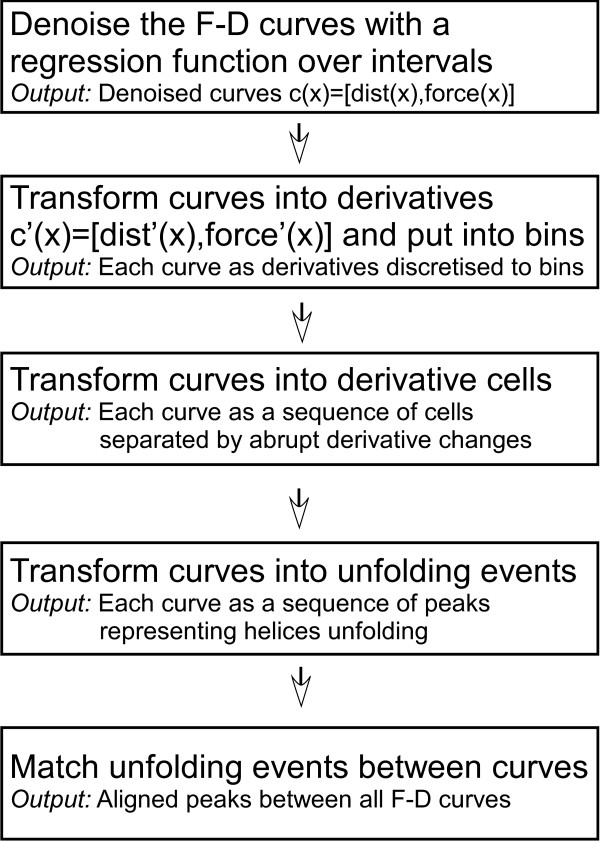
**The flow of the analysis procedure: First, F-D curves are denoised**. Next, we transform the curves to derivatives that represent increasing, decreasing, or constant force. Next, we detect the unfolding events as peaks in the curves. An alignment procedure matches peaks between curves that correspond to the same unfolding event. The unfolding patterns are constructed this way, by matching corresponding unfolding events between curves.

Figure 3 provides an overview of the steps of our procedure for finding unfolding patterns.

### 3.1 Step 1: denoise the F-D curves

**Figure 4:**
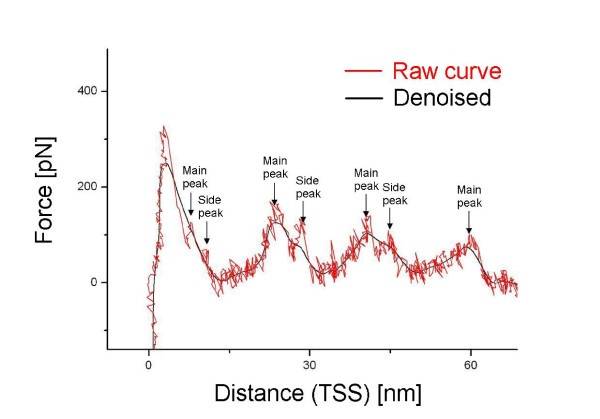
**A raw F-D curve before and after denoising**. The main peaks and side peaks are shown along the F-D curve.

The F-D curves are usually noisy, which hinders our aim to detect peaks. Before applying our algorithm on the dataset, we denoise each SMFS curve. Each curve is modeled as a 2D parametric curve *c*(*x*) = [*dist*(*x*), *force*(*x*)], where *x *represents the timeline of the pulling experiment that produced the F-D curve. First, we apply regression to remove the global noise at a large-scale; each of *dist*(*x*), *force*(*x*) is independently denoised using robust locally weighed scatter plot smoothing and least squares linear polynomial fitting (RLOWESS) [23]. Figure 4 shows an SMFS curve before and after denoising. We tried several denoising intervals, such as 11, 51 and 101 data points. With a raw F-D curve consisting of ~ 1, 600 data points, we selected denoising interval of 51 points. The reason is we expect from the protein structure to observe 3 main unfolding events (peaks) and several side peaks; while 11 and 101 points gave too many or too few unfolding events, 51 points gave the expected number of events. Subsequently, we interpolated each denoised curve to a representation consisting of 50, 000 data points.

### 3.2 Step 2: find the derivatives of the F-D curves

**Figure 5:**
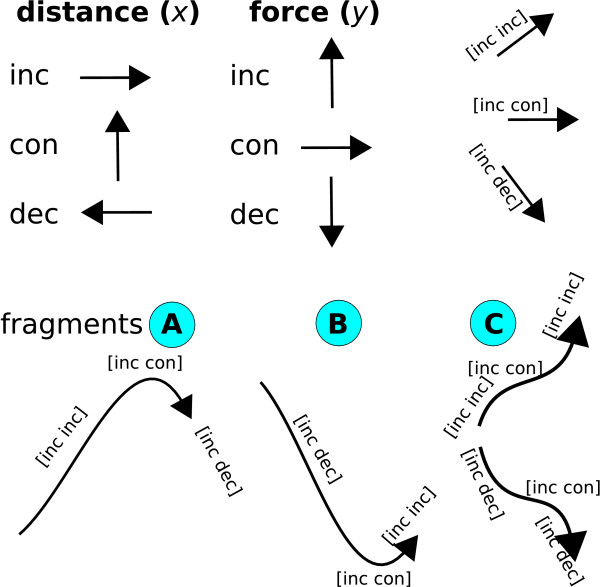
**Top: In an F-D curve, the distance (tip sample separation) may be increasing or constant along the *x*-axis**. The force may be increasing or decreasing or constant along the *y*-axis. A point in the F-D curve can be described as a pair, describing the changes of distance and force, as shown in brackets. To determine the changes along *x *and *y *axes, we get the derivatives and we discretise them. Bottom: An F-D curve can be described as a sequence of fragments describing the changes. Fragment *A *is local maximum force, which may be a main or side peak in the F-D curve. Fragment *B *is local minimum force, which separates two unfolding events in the F-D curve. Fragment *C *is increasing or decreasing force interrupted by a cliff of constant force, which may be a side peak in the F-D curve.

Figure 5 shows how we convert each F-D curve representation from Step 1 to a sequence of derivatives. The derivatives show how the curve changes relative to the distance (*x*-axis) and the force (*y*-axis). The derivatives are then further discretised into bins (cells) based on whether they are increasing, decreasing, or remain constant. We describe an F-D curve as a sequence of fragments that may be of three types, named A, B, or C; these fragments represent changes of distance and force in the F-D curve.

To get the derivatives we deal with each F-D as an arc length parameterised curve *c*(*x*) = [*dist*(*x*), *force*(*x*)], such that , which implies , which implies |*dist*'(*x*)| ≤ 1 and |*force*'(*x*)| ≤ 1. In other words, arc length parameterised curves do not change abruptly, implying that this parameterisation makes it feasible for us to discretise the space of derivatives, since all derivative values will be in the range [-1 ⋯ 1].

Without such a bound on the space of derivatives this approach would run into problems, since it would be difficult to appropriately discretise a curve.

We discretise the space of derivatives for the *x*-axis (distance) and *y*-axis (force) into 1, 000 bins. We then represent the curve as a sequence of tuples (*dx_i_*, *dy_i_*), each of which denotes the current *derivative cell *in which the curve is located. A new tuple (*dx_i_*, *dy_i_*) is added to the sequence of tuples whenever the curve's derivative changes significantly enough to warrant a new derivative cell (Figure 5). Therefore, a linear curve would be encoded by a single derivative cell, since its slope is constant.

With each derivative cell we also associate the arc length (distance) in the denoised curve that the cell covers. The arc length of a curve can be thought of as the "length" of a piece of string if it were laid upon the curve. Let *t *be the absolute length of a F-D curve segment - this is the length of a string if it was laid along the F-D curve segment. We use the arc length to ignore any cells that cover small F-D curve segments, as determind by a minimum threshold *t_small_*. The arc length of a curve *c*(*x*) from point *t*_0 _to *t *is defined to be , where |*c*'(*x*)| is the norm of the vector *c*'(*x*).

#### 3.2.1 Translational Invariance

**Figure 6:**
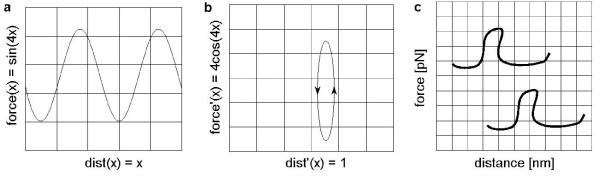
**The motivation for discretising the derivative spaces of F-D curves is translational invariance, allowing us to find similar patterns of change in F-D curves that are translated with respect to each other**. (*a*) The function *c*(*x*) = (*x*, *sin*(4*x*)) may fit a hypothetical path in an F-D curve. (*b*) The derivative space *c*'(*x*) = (1, 4 *cos*(4*x*)). (*c*) Hypothetical paths in two F-D curves that are translated with respect to each other will look similar in derivative space.

Figure 6 shows examples of F-D curves that are translated with respect to each other. Assume *c*_1_(*x*) = [*dist*(*x*), *force*(*x*)] and *c*_2_(*x*) = [*dist*(*x*) + 5, *force*(*x*) + 3]. In other words, *c*_2 _is a translated version of path *c*_1_. If we take the derivatives  of these two paths, then, we notice that  for all values of *x*. We use this fact to mine F-D curves that are translated with respect to each other on the basis of their derivative changes. The F-D curve mining is invariant to the unknown amount by which the curve was translated by the SMFS machine. Note, that to get the derivatives, we assume that we are dealing with differentiable functions that do not have abrupt edges. Another issue to keep in mind is that derivatives are sensitive to noise. Therefore, denoising (step 1) is essential for dealing with this issue.

### 3.3 Step 3: unfolding events

**Figure 7:**
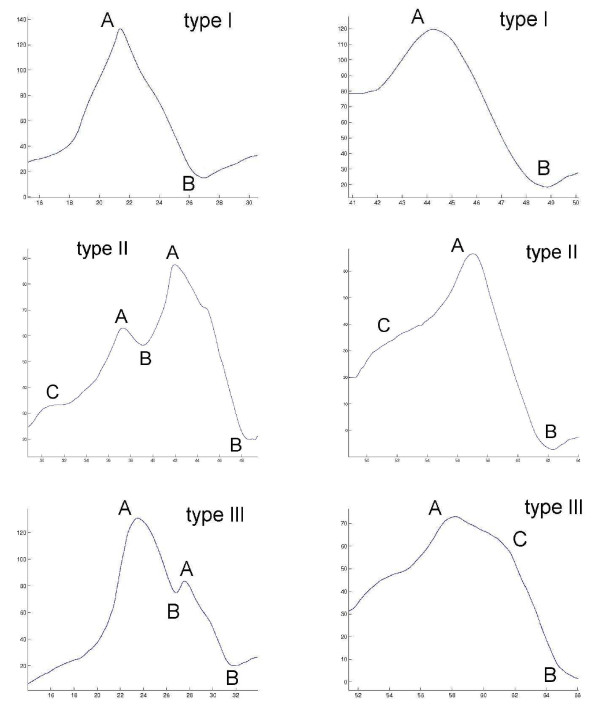
**We describe an entire SMFS curve as a sequence of fragment types: *A *is a local maximum, *B *is a local minimum, and *C *is a cliff**. Unfolding events in SMFS curves are categorised in three types: *I*. Main peak, where two bR helices unfold together. *II*. Main peak preceded by a side peak, where the helices unfold stepwise, one after another. *III*. Main peak followed by a side peak, where one helix unfolds gradually, and then another helix in an all-or-none manner. The events are matched to one another between the curves to detect corresponding unfolding. On the unfolding events one can fit the Worm-Like Chain model (WLC) for polymer stretching. In turn, one can compute the delta-distances (in amino acids) between the WLCs and view histograms of delta-distances.

Figure 7 shows that sequences of A, B, or C fragments in an F-D curve can describe several types of unfolding events. Type I unfolding event is a main peak "AB" without side peaks. The other two events include side peaks before or after the main peak. Type II unfolding event is a main peak "CAB", where the side peak is "CA". Type III unfolding event is a main peak "ACB", where the side peak is "AC". After finding a peak, one can fit the Worm-Like Chain model to the peak. Since a WLC maps to a specific amino acid of the protein sequence, a WLC allows one to map an unfolding event to the protein sequence and/or structure. The protein structure can be colored in 3D (using Jmol) to reflect the helices the unfolding of which corresponds to a WLC peak.

### 3.4 Step 4: matching unfolding events between curves

Step 4 supports finding patterns of unfolding events in the F-D curves, rather than simple peaks. To describe the unfolding patterns of the F-D curves we match the unfolding events between curves [8]. For this purpose we use a progressive alignment, the aim of which is to align the F-D curves by a pairwise matching of detected unfolding events [24]. Unfolding events are matched between F-D curves if they likely correspond to the same helices unfolding.

**Figure 8:**
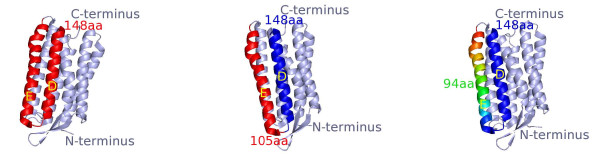
**The unfolding events (peaks) are matched between F-D curves if they correspond to the same helices unfolding**. *Left *: Helices E and D unfold in a single step. The polypeptide chain extending between the AFM cantilever tip and surface exhibits a length of 148 aa (tip-sample separation of ~ 53 *nm*). *Middle *: Helices E and D unfold in a two-step process. First, helix E unfolds with the polypeptide chain lengthened to 105 aa (TSS of ~ 38 *nm*). Second, helix D unfolds with the polypeptide chain lengthened to 148 aa (TSS of ~ 53 *nm*). *Right *: Similar to *middle*, except first helix E unfolds partly, with the polypeptide chain lengthened to 94 aa (TSS of ~ 34 *nm*) [5]. Matched unfolding events (peaks) are within a window of 5 amino acids (~ 2 *nm*) from each other, as indicated by the tip-sample separation at the end of the peak (1*aa *≈ 0.36 *nm*). An entire F-D curve is shifted by a terminal length of at most 30 amino acids, which results in the most matches; the terminal length represents the location of cantilever attachment to the protein.

Assume a curve C, which is presented to a set of previously aligned curves A. The scores for the matches/mismatches are chosen in the following way:

The score for aligning the unfolding events in C with A is the sum over all match/mismatch scores of matched events between C and A. A match is assumed, if the distance between event *p *∈ *C *and *p*' ∈ *A *is less than 5 amino acids. Figure 8 shows three examples of helix unfolding events in bR, which are within a distance of 5 amino acids from one another. All unfolding events in a F-D curve can be shifted by a maximum number of 30 amino acids, accounting for the location of cantilever attachment on the C- or N-terminus of the protein sequence. The shift S of all unfolding events in curve C is found, which results in the best alignment score for C and A.

#### 3.4.1 Main peaks and side peaks

The alignment allows matching unfolding events between curves. After the alignment, we represent an F-D curve as a sequence of (0, 1) signs, corresponding to whether or not an event occurs. A possible event is represented by a sign of (0, 1). All F-D curves have the same maximum number of possible events. The curve alignment on the basis of the detected events allows to find the unfolding pathways for bR.

By examining the frequency of an event over all curves we categorise it as a main peak or side peak. A peak with highest frequency is a main peak, while peaks of lower frequency are side peaks. It is possible for both a side and main peak to be found in an unfolding event of a curve, in which case the side peak is the cliff before or after the main peak ("CAB" or "ACB" in Figure 7).

## 4 Results and Discussion

Our goal is to find the different unfolding pathways of the bR membrane protein. To this end, we use our algorithm to detect the unfolding events and align them between F-D curves, as described above. Table 1 shows the manually curated sequences of 0 or 1 for three helix pairs in the 26 bR curves [22]. As shown, for each helix pair the unfolding pattern consists of a main peak and possibly one or two side peaks.

**Table 1:** Unfolding of transmembrane helices in bR results in different unfolding pathways.

Region 1 (Helices E&D)	Region 2 (Helices B&C)	Region 3 (Helix A)	Unfolding pathways
(1 0 0)	(1 0 0)		100 100 10/11
(1 1 0)	(1 1 0)	(1 0)	100 110 10/11
(1 0 1)	(1 0 1)	(1 1)	100 101 10/11
(1 1 1)	(1 1 1)		100 111 10/11

		Total	8

The table shows the different unfolding pathways that are observable in the membrane protein bR. Sign "1" represents the presence of an event in the corresponding region, while "0" means no event. The analysis leads to 8 different unfolding pathways. The first unfolding pathway is given by the pattern 100 100 10, the second by the pattern 100 100 11, which means that in the third region we have two peaks, corresponding to the stepwise unfolding of helix A.

Our goal is to evaluate how well the main and side peaks that our algorithm detected correspond to this manual curation. For this purpose, we evaluated over the aligned curves how many of the detected peaks correspond to the manually curated peaks. Tables 2 and 3 show 3 main peaks and 4 side peaks, respectively, which we detected in various regions of the bR curves. For each of the 26 bR curves, we analyzed which of the peak detections were *TP *true positive or *FP *false positive peaks. With *TP *= 83, *FP *= 9, clearly *TP >> FP*, implying a high success rate. The last side peak at 232aa [22] was missed in our results, which is due to noise in this region. It is possible to detect this last peak by relaxing the minimum threshold *t_small _*for the arc length, but the tradeoff is an increase in the number of FP peaks.

**Table 2:** The co-occurrences of all main peaks in the curves.

Main peak		Co-occurrence frequency (out of 26 curves)
**Region**	**contour length [aa]**	

1	80	15
2	143	24
3	215	26

1 & 2	80 & 143	14
1 & 3	80 & 215	15
2 & 3	143 & 215	24

1 & 2 & 3	80 & 143 & 215	14

**Figure 9:**
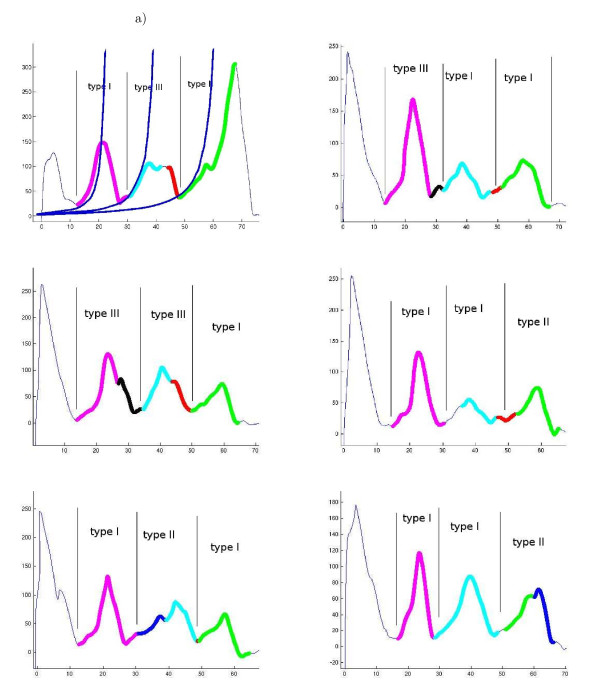
**In bR there are three main unfolding events, which are detected in F-D curves as main peaks**. Each unfolding event corresponds to an unfolding of helices in the bR structure. *a*) This figure shows the Worm-Like Chain model fit to the peak, which allows one to map the unfolding event to a specific amino acid in the structure [13,20,22]. The three main peaks appear in most curves and have a high co-occurrence with one another. However, the unfolding pathways are defined by the side peaks that occur in a minority of curves.

The co-occurrences of these main peaks in the same curve were high. The contour length comes from the fitting of the Worm-Like Chain model inside the curves (see Figure 9a) and it corresponds to knowledge from the literature [13,20,22].

**Table 3:** The side peaks do not co-occur frequently in the same curves.

Side peak		Co-occurrence frequency (out of 26 curves)
**Region**	**contour length [aa]**	

1	39	9
1	97	4
2	167	10
3	201	4

1 & 1	39 & 97	2
1 & 2	39 & 167	1
1 & 2	97 & 167	2
1 & 3	97 & 201	1
2 & 3	167 & 201	1

1 & 2 & 3	39 & 97 & 167 & 201	0

Yet, most of the side peaks occur individually much more frequently in curves. The contour length comes from the fitting of the Worm-Like Chain model inside the curves (see Figure 9a) and it corresponds to knowledge from the literature [13,20,22].

### 4.1 Matching unfolding events in F-D curves

Figure 9 shows six F-D curves. In this example, the three main peaks that are matched in the curves are colored similarly. These peaks correspond to the pairwise unfolding of transmembrane helices in bR [13]. Side peaks have special colors and occur less frequently than main peaks. The side peaks correspond to intermediate states in the unfolding process, meaning that the helices unfolded one after the other with an intermediate step, instead of pairwise. This makes it interesting to study the co-occurrences of main and side and main/side peaks within bR curves.

Our analysis provides several advantages over simply detecting minima in the derivatives of the smoothed force curves. After matching unfolding events in all included F-D curves, it is possible to fit the WLC model, as Figure 9a shows. The tables show the contour lenghts. Besides computing the contour lengths of the WLCs, we can also distinguish the different unfolding pathways directly in the process. The unfolding pathways we find give hints on the stability inside proteins. Moreover, we can compare the wildtype protein's unfolding pathways with mutants of the protein under study, or we can study the effect of a ligand.

### 4.2 Side peaks: co-occurrences analysis

The main peaks appear in most of the included F-D curves and have a relatively high co-occurrence with one another in the curves. However, the different unfolding pathways are defined by the side peaks that occur in a minority of curves. Different co-occurrences are observed for various main and side peak pairs, which define the unfolding pathways. The helices in transmembrane proteins often stabilise one another. Intermediate side peaks between main peaks reflect stepwise unfolding of helix pairs and helices alone, such as helices E and D, or B and C [25-27].

Table 2 shows that the main peaks frequently co-occur with one another in F-D curves.

Table 3 shows that the side peaks co-occur less frequently with one another.

Table 4 shows that the side peaks nearly always co-occur with at least one main peak. This implies a synergistic effect occurring between helices. Two helices unfolding stepwise with an intermediate step (detected as a side peak) may stabilise another pair of helices, resulting in pairwise unfolding. In those cases where a side peak occurs before the main peak there is a helix unfolding gradually step-by-step, and then a helix unfolds in an all-or-none manner [14,15]. For example, helices F and G neighbor helices A and B and the former may stabilise the latter. Then, an intermediate unfolding step may be observed for helices F and G.

**Table 4:** The co-occurrences of side peaks and main peaks in curves.

Side & Main peak		Co-occurrence frequency (out of 26 curves)
**Region**	**contour length [aa]**	

1 & 1	39 & 80	6
1 & 2	39 & 143	8
1 & 1	97 & 80	2
1 & 2	97 & 143	4
2 & 2	167 & 143	10
2 & 3	167 & 215	10
3 & 2	201 & 143	4
3 & 3	201 & 215	4

As shown, the side peaks nearly always co-occur with a nearby main peak in the same curve. Moreover, the first two side peaks (39aa, 97aa) co-occur more often with the second main peak (143aa) than with the first main peak (80aa). The main peak at 80aa also occurs, overall, less frequently in curves. The contour length comes from the fitting of the Worm-Like Chain model inside the curves (see Figure 9a) and it corresponds to knowledge from the literature [13,20,22].

We have also analyzed four bR mutants, as well as the ompG protein with this algorithm [28]. Even though the mutant proteins are known to have different unfolding patterns, we could detect the known unfolding events. Our results for mutant proteins corresponded to the results of Sapra et al. [20,22]

### 4.3 Comparison to previous methods and runtime

Our method has similar precision and recall to the method published previously by Marsico et al. [19] However, our algorithm has the advantage of faster detection of protein unfolding patterns. For the 26 bR curves the method by Marsico et al. took several hours. Our method's total runtime for denoising, getting the derivatives, discretising, detecting the unfolding events and aligning the 26 curves was less than one second.

Moreover, we attempted to evaluate Punias [10] and Hooke [12] on the manually annotated bR dataset. These algorithms focus on fitting the Worm-like Chain model on F-D curves in an automated fashion, and do not focus on finding the unfolding pathways as our algorithm; therefore a complete comparison cannot be done. On fitting the WLC on the manually annotated bR dataset, Punias achieved 79% precision, 53% recall and 64% F-measure. Hooke achieved 73% precision, 45% recall and 56% F-measure. These results indicate that our method is at least as effective as Punias and Hooke.

## 5 Conclusions

Single-molecule force spectroscopy is a promising method for measuring the unfolding forces of single molecules and cells. SMFS can analyze membrane proteins in their natural membrane environment. Our main contribution is a novel method for analyzing and classifying SMFS data. Our pattern recognition algorithm is successful in finding unfolding pathways of bR. Our method for finding unfolding events and alignment is much faster than a manual selection and annotation. With our automated approach, the detection of unfolding events is not subjective to the manual annotator, but rather is based on objective criteria. Overall, our algorithm gives a high success rate in observation of bR unfolding pathways. The method also has the advantages of discovering side and main peaks along with unfolding patterns, fitting the WLC model on the peaks, and computing the amino acid distances between contour lengths. As future work, we plan to link the unfolding events to structural features, such as residue-residue contacts and membrane topology.

## Competing interests

The authors declare that they have no competing interests.

## Authors' contributions

BA conceptualised and implemented the methods, performed the experiments and wrote most of the paper. DL provided the datasets and supervised the work and the development of ideas. All authors read and approved the final manuscript.
